# Supplementary material for: A Comparative Elemental Analysis of Espresso Coffee from Poland and Portugal
Source: Foods. 2025 Jan 28;14(3):426. doi: 10.3390/foods14030426 (PMC11817413; doi:10.3390/foods14030426)
Supplement: Supplementary file 1 [file foods-14-00426-s001.zip › foods-3384168-supplementary.pdf]

**Table S1.** Concentration (µg/L) of macro- and trace elements in coffee samples from Poland and Portugal.

| Elements | Poland | | | Portugal | | | p** |
|---|---|---|---|---|---|---|---|
| | P25 | Median | P75 | P25 | Median | P75 | |
| **B** | 1084 | 1545 | 2133 | 971 | 1234 | 1503 | 0.003 |
| **Mg** | 186177 | 242876 | 344148 | 130883 | 171236 | 195150 | < 0.001 |
| **P** | 147339 | 210550 | 273336 | 142396 | 166826 | 200258 | 0.014 |
| **Ca** | 63060 | 80760 | 108880 | 38462 | 52189 | 60950 | < 0.001 |
| **Mn** | 1173 | 1713 | 2762 | 658 | 815 | 1118 | < 0.001 |
| **Ni** | 45.3 | 81.0 | 133 | 58.0 | 86.8 | 121 | 0.747 |
| **Cu** | 112 | 552 | 1220 | 72.1 | 178 | 492 | 0.003 |
| **Zn** | 336 | 660 | 1202 | 156 | 276 | 557 | < 0.001 |
| **As** | < 2.78 | < 2.78 | < 2.78 | < 2.78 | 3.18 | 4.60 | -* |
| **Se** | < 2.78 | < 2.78 | < 2.78 | < 2.78 | < 2.78 | < 2.78 | -* |
| **Rb** | 3913 | 4984 | 6671 | 3460 | 4665 | 6453 | 0.389 |
| **Sr** | 206 | 273 | 376 | 134 | 189 | 265 | < 0.001 |
| **Mo** | 1.28 | 2.56 | 4.27 | 0.889 | 1.97 | 4.14 | 0.252 |
| **Cd** | < 0.068 | 0.202 | 0.396 | < 0.068 | 0.096 | 0.182 | 0.004 |
| **Sn** | 0.173 | 0.442 | 1.65 | 0.320 | 1.27 | 4.22 | 0.054 |
| **Sb** | < 0.044 | 0.103 | 0.176 | 0.099 | 0.124 | 0.160 | 0.322 |
| **Cs** | 5.83 | 8.44 | 16.5 | 8.00 | 12.4 | 17.2 | 0.129 |
| **Ba** | 96.6 | 146 | 229 | 51.2 | 79.8 | 108 | < 0.001 |
| **Tl** | < 0.026 | 0.053 | 0.091 | < 0.026 | 0.059 | 0.088 | 0.623 |
| **Pb** | 2.49 | 14.3 | 34.0 | 1.99 | 4.43 | 15.6 | 0.039 |

*Not Included because most of the data was < LD; ** non-parametric Mann-Whitney test.

**Table S2.** Concentration (µg/L) of macro- and trace elements in water samples from Poland and Portugal.

| Elements | Poland | | | Portugal | | | p** |
|---|---|---|---|---|---|---|---|
| | P25 | Median | P75 | P25 | Median | P75 | |
| **B** | 59.2 | 105 | 211 | 4.93 | 6.97 | 11.0 | < 0.001 |
| **Mg** | 9188 | 10722 | 12742 | 4472 | 5038 | 5857 | < 0.001 |
| **P** | 110 | 127 | 297 | 26.1 | 88.4 | 173 | 0.007 |
| **Ca** | 64933 | 76671 | 90053 | 20165 | 26722 | 30405 | < 0.001 |
| **Mn** | 1.97 | 3.31 | 6.72 | 0.715 | 1.91 | 3.74 | < 0.001 |
| **Ni** | 4.08 | 5.58 | 9.62 | 1.09 | 4.60 | 6.59 | 0.022 |
| **Cu** | 27.1 | 64.2 | 127 | 12.0 | 20.5 | 41.2 | < 0.001 |
| **Zn** | 69.5 | 187 | 470 | 29.9 | 63.1 | 128 | < 0.001 |
| **As** | < 2.78 | < 2.78 | < 2.78 | < 2.78 | < 2.78 | 3.89 | -* |
| **Se** | < 2.78 | < 2.78 | < 2.78 | < 2.78 | < 2.78 | < 2.78 | -* |
| **Rb** | 1.15 | 1.62 | 2.43 | 1.44 | 1.70 | 1.83 | 0.708 |
| **Sr** | 224 | 274 | 567 | 138 | 178 | 183 | < 0.001 |
| **Mo** | 0.832 | 1.18 | 1.71 | 0.233 | 0.417 | 0.545 | < 0.001 |
| **Cd** | < 0.068 | 0.072 | 0.135 | < 0.068 | < 0.068 | < 0.068 | < 0.001 |
| **Sn** | 13 | 0.155 | 0.749 | < 0.056 | 0.110 | 0.247 | 0.002 |
| **Sb** | < 0.044 | 0.057 | 0.116 | 0.131 | 0.147 | 0.162 | < 0.001 |
| **Cs** | < 0.055 | < 0.055 | 0.065 | < 0.055 | < 0.055 | < 0.055 | -* |
| **Ba** | 19.1 | 23.7 | 36.8 | 14.5 | 17.6 | 20.4 | < 0.001 |
| **Tl** | < 0.026 | < 0.026 | 0.031 | < 0.026 | < 0.026 | < 0.026 | -* |
| **Pb** | 1.58 | 2.15 | 3.89 | 0.254 | 0.574 | 1.37 | < 0.001 |

*Not Included because most of the data was < LD; ** non-parametric Mann-Whitney test.

**Table S3.** Contribution (%) of water to the concentration of macro- and trace elements in coffee espresso samples from Poland and Portugal.

| **Elements** | **Poland** | | | **Portugal** | | |
|---|---|---|---|---|---|---|
| | **P25** | **Median** | **P75** | **P25** | **Median** | **P75** |
| **B** | 4.7% | 7.3% | 15% | 0.4% | 0.6% | 1.1% |
| **Mg** | 3.3% | 4.8% | 6.3% | 2.2% | 3.3% | 4.0% |
| **P** | 0.05% | 0.08% | 0.13% | 0.06% | 0.02% | 0.11% |
| **Ca** | 55% | 92% | 127% | 39% | 54% | 70% |
| **Mn** | 0.11% | 0.21% | 0.56% | 0.08% | 0.23% | 0.51% |
| **Ni** | 4.7% | 7.1% | 17% | 1.4% | 5.0% | 7.4% |
| **Cu** | 3.6% | 14% | 46% | 5.0% | 10% | 34% |
| **Zn** | 11% | 27% | 80% | 8.1% | 24% | 73% |
| **Rb** | 0.02% | 0.03% | 0.06% | 0.03% | 0.04% | 0.05% |
| **Sr** | 71% | 110% | 229% | 48% | 85% | 117% |
| **Mo** | 32% | 60% | 110% | 8.8% | 14% | 26% |
| **Cd** | 23% | 36% | 72% | 29% | 47% | 52% |
| **Sn** | 13% | 35% | 121% | 1.0% | 20% | 65% |
| **Cs** | 0.27% | 0.59% | 1.0% | 0.25% | 0.37% | 0.71% |
| **Ba** | 11% | 17% | 35% | 13% | 22% | 35% |
| **Pb** | 7.5% | 21% | 101% | 3.2% | 10% | 23% |

The elements As, Se, Sb and Tl were not included because most of the data was < LD.
